# CoPc/rGO and CuPc/rGO Nanocomposites: Comparative Characterization and Study of Their Gas Sensing Properties

**DOI:** 10.1021/acs.inorgchem.5c01085

**Published:** 2025-06-26

**Authors:** Çiğdem Yağcı, Oluwatoyin Emmanuel Aina, Atefeh Emami, Amal Ibijbijen, Diana Dragoe, Haifa Ben Aziza, Ümit İşci, Abdelhamid Errachid, Nicole Jaffrezic Renault, Fabienne Dumoulin, Hafsa Korri-Youssoufi

**Affiliations:** † ICMMO, UMR-CNRS, Université Paris-Saclay, 17 avenue des sciences, Orsay 91405, France; ‡ Faculty of Education, Umuttepe Campus, 52980 Kocaeli University, İzmit, Kocaeli 41001, Türkiye; § CNRS, ISA-UMR 5280, CNRS, 27098 Université Claude Bernard Lyon 1, 5 Rue de la Doua, Villeurbanne 69100, France; ∥ Faculty of Engineering and Natural Sciences, Biomedical Engineering Department, Acıbadem Mehmet Ali Aydınlar University, Ataşehir, Istanbul 34752, Türkiye; ⊥ Department of Metallurgical and Materials Engineering, 52982 Marmara University Faculty of Technology, Maltepe, Istanbul 34722, Türkiye; # UTINAM Institute, University of Franche-Comte, 16 Gray Road, Besancon 25030, France

## Abstract

Nanocomposites made of tetrahexyloxy-substituted Co or Cu phthalocyanines adsorbed onto reduced graphene oxide (**rGO**) have been prepared for detection of volatile organic compounds (VOCs). Such nanocomposites could be formed in organic solvents, thanks to the high solubility of the substituted phthalocyanines, establishing van der Waals and π–π stacking interactions with **rGO**. UV–visible, FT-IR, and XPS characterizations evidenced the energy transfer between **rGO** and the phthalocyanines; this adsorption ability is highly suitable to form nanocomposites exhibiting improved electronic properties. The gas sensing properties of various VOCs were explored by conductometric measurements conducted on interdigitated electrodes modified with the nanocomposites, highlighting the effect of the alkyloxy substituents and phthalocyanine’s metal. All exhibited high affinity to ammonia gas compared with other VOCs. The **CuPc/rGO** nanocomposites showed higher sensitivity with a lower limit of detection (0.4 ppm) compared to **CoPc/rGO** (1 ppm), while phthalocyanines alone give lower sensitivity, where the limit of detection of **CuPc­(OHex)**_**4**_ is 5.7 ppm and **CoPc­(OHex)**_**4**_ shows a nonlinear fit. We explained this behavior by improved energy transfer between **rGO** and **CuPc­(OHex)**_**4**_, favoring the coordination of ammonia. Detection of ammonia gas in real samples has been achieved to further demonstrate the efficiency of these sensors.

## Graphical abstract

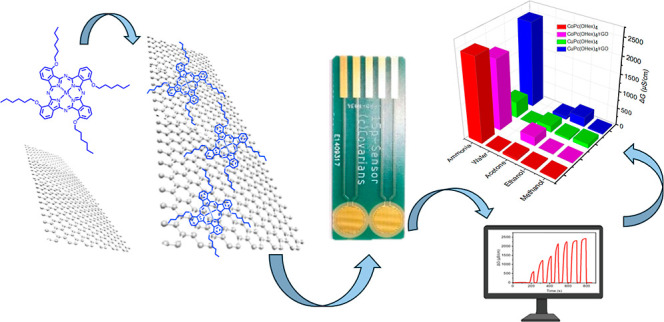

## 1 Introduction

Gas sensing plays a crucial role in public health management by monitoring air quality and detecting harmful gases that can affect human health and the environment. These sensors help identify the presence of toxic gases such as carbon monoxide (CO), nitrogen dioxide (NO_2_), sulfur dioxide (SO_2_), and ammonia (NH_3_), all gases that are often released from industrial activities and vehicle emissions, and are also present in households.^1^ By providing real-time data, gas sensors enable early warning systems, helping to prevent health risks such as respiratory diseases, cardiovascular problems, and even long-term conditions caused by exposure to poor air quality. One of the most toxic gases is ammonia (NH_3_). Harmful exposure can cause urinary tract infections and burn the mucous membranes of the eyes and respiratory organs.^2^ Ammonia is commonly released from agriculture, industrial processes, and wastes. Detecting ammonia helps protect workers, communities, and urban environments by enabling quick responses to leaks, ensuring safety, and improving air quality management. Various sensors were developed for gas sensing based on metal oxides^3^ or semiconductors.^4^ Organic semiconducting materials are increasingly used due to their unique properties as π-conjugated materials that can play the role of a receptor and transducer.^5^

Phthalocyanines (Pcs) are very stable and usually planar molecules.^6^ Properties of metalated Pcs (MPcs) are influenced by the type of central metal ion and the macrocyclic substitution,^7^ axial ligands when applicable,^8^ and microenvironment.^9^ When appropriately designed, Pcs exhibit properties especially suitable for use as organic semiconducting materials^10^ in electronics,^11^ photosensitizers for photodynamic therapy,^12^ sensors,^13^ catalysis,^14^ and photovoltaic^15^ applications. Pcs can coordinate many metal ions such as zinc, copper, cobalt—among others—to improve their electron transfer ability.^16^ Their maximum absorption band (known as the Q-band) in the electronic spectra is significantly influenced by the presence of electron-donating or electron-withdrawing groups on the Pc core.^17^ The introduction of electron-donating alkoxy substituents onto the Pc core leads to improved chemical and physical properties,^18^ primarily their solubility,^19^ and prevents their aggregation, which usually has a detrimental effect on the targeted properties.^20^ Thanks to their planarity, Pcs possess surface/interface-related features that are essential for many applications^21^ and favor their adsorption onto various materials.^22^ Their strong π-conjugation makes Pcs highly sensitive to changes in electron density^23^ and may enhance their selectivity and sensitivity to a wide range of analytes, including gases such as volatile organic compounds (VOCs), environmental pollutants, and toxic gases such as ammonia^24^ or carbon monoxide.^25^

On the other hand, intense research on graphene and other 2D materials has quickly developed over the last decades. Graphene, one of the advanced carbon nanomaterials, is a 2-dimensional single sheet of carbon atoms with a honeycomb lattice structure, and it is at the origin of graphitic materials of all other dimensions (0D fullerenes, 1D nanotubes, and 3D graphite). Currently, graphene-based emerging 2D nanomaterials have been attracting tremendous attention and interest because of their unique physicochemical properties,^26^ high surface area to volume ratio (theoretically 2630 m^2^/g for single-layer graphene), excellent thermal and electric conductivity, and strong mechanical strength.^27,28^ These properties prompt the use of graphene devices in various practical biotechnology applications including catalysts,^29^ electrochemical energy storage,^30^ fuel cells,^31^ lithium and sodium batteries,^32^ sensors,^33^ biosensors,^34^ among others. The electronic properties of graphene sheets early prompted their use as electrodes for electrochemistry and electrocatalysis. Sensing platform*s* with electrical or electrochemical readout based on graphene and graphene nanocomposites have been developed for the detection of several samples such as biomolecules or organic molecules and VOCs.^35^ Graphene as a transducer for electrochemical or electrical sensors is full of promise due to its simplicity and highest sensitivity of detection,^36^ more often coupled with low frequency 1/f noise^37^ and low electron transfer resistance.^38^ Reduced graphene oxide (**rGO**) is a graphene-like 2D derivative obtained by the reduction of graphene by various techniques.^39^ The resulting material usually has some defects affecting negatively its conductivity^40^ but positively its electron transfer ability.^41^ In recent years, the integration of π-conjugated macrocyclic complexes, such as Pcs, with graphene-based materials has emerged as a promising strategy to design hybrid systems for electronic and sensing applications. rGO is indeed hydrophobic and tends to agglomerate in water through strong π–π stacking and van der Waals interactions between graphene sheets. As a consequence, considerable efforts are being made to improve its dispersion by the entrapment of functional molecules, which allows better dispersibility in organic solvents.^42^ **rGO**-based electrochemical sensors have demonstrated a large domain for target molecules binding but also an effective electron transfer ability and signal amplification.^43^

The association of graphene with macromolecules such as semiconducting MPcs presents several advantages, such as improving the electronic properties of the nanocomposites by limiting the stacking and improving their dispersion, hence the surface to volume ratio,^44^ conferring enhanced electrochemical and electronic properties, and optimizing electron transfer ability.^45^ The association could be performed by covalent attachment^46^ or by π–π stacking interaction, leading to modification of chemical properties of **rGO** or of the Pc.^47^ The covalent attachment affects the sp^2^-hybridized carbon atoms of **rGO**, leading to a defect in the structure due to the presence of the sp^3^ structure. In the case of the interaction through π–π stacking with the core of Pc, it modifies the chemical properties of the dye and the accessibility of the core for further reaction.^48^ However, significant challenges remain, notably, the poor solubility of classical Pcs and the limited scalability of composite fabrication methods.

The association of graphene with MPcs has been demonstrated to be a good way to improve gas sensing selectivity and sensitivity.^49^ This is due to availability of various substituted groups,^25c^ shape of the Pc when it has axial substituents in the case of widely used SiPcs,^48b^ and metal ions in MPcs^50^ that contribute to improving the specific gas interaction. In addition, the high surface area of graphene favors various sites of association, improving the sensitivity of the detection. The detection relies generally on monitoring the electronic properties of the nanocomposites and measuring the variations of conductometry or chemiresistivity. For example, hexadecafluorinated copper pPc (F_16_CuPc) associated with **rGO** was demonstrated to detect ppb levels of chlorine gas at room temperature.^51^ A nanocomposite formed with a tetra-(fluoromethylphenoxy) substituted CoPc and **rGO** demonstrated high-performance ppb-level hydrogen sulfide sensing at room temperature.^52^ However, in the case of ammonia detection, only a few studies have demonstrated the effects of the substituents and metal ions in the detection process. MPcs modified with phenyloxy alkyl groups with various metals in association with **rGO** were studied for ammonia detection and demonstrated that the factor governing the sensitivity of the detection to ammonia is the electrical properties of the nanocomposite and their surface area.^53^

Here, we explore the association of **rGO** and substituted soluble MPcs by van der Waals interactions, with the aim of improving the chemical and electrical properties of the Pcs to be available for further coordination with other compounds, such as VOCs. For this purpose, we designed and synthesized Cu and Co Pcs bearing four hexyloxy alkyl chains in nonperipheral positions to make them highly soluble in organic media. Next, **rGO** was synthesized by the modified Hummers^54^ method, reduced by the solvothermal method, and used to form nanocomposites. The properties of the obtained nanocomposites were studied to highlight the effect of MPcs on the structural and electronic properties of **rGO**. Finally, gas sensing experiments conducted on these nanocomposites by following the conductivity variation of the nanohybrids using conductometric measurement were performed. The properties of the **CuPc/rGO** and **CoPc/rGO** nanocomposites were studied by UV–visible, FT-IR, XPS, and electrochemical methods to demonstrate the effect of Pc on the morphological, structural, electronic, and electrochemical properties of the nanocomposite and to highlight the charge transfer. The application of these nanomaterials in gas sensing was performed with the detection of ammonia, methanol, ethanol, and acetone. We demonstrate the contribution of the molecular structure of the Pc and the effect of the metal core of the composite on sensitivity and selectivity in VOC detection. These works are original and contribute to the field to various extents: we introduce a new class of **MPc/rGO** nanocomposites fabricated with novel soluble alkyloxy-substituted pPcs. These substituents advantageously improve the Pc solubility in organic media and modulate their interfacial electronic interactions with **rGO**. An original synthesis approach for the preparation of the nanocomposites combines ultrasonication and purification by centrifugation, thereby enabling scalable production of the composites, ensuring full utilization of the graphene sheets. These structural and electronic improvements translate into enhanced gas sensing performance, particularly in ammonia detection, compared to previously reported MPc/rGO assemblies.

## 2 Experimental Section

### 2.1 Reagents

Graphite, NaNO_3_, H_2_SO_4_, KMnO_4_, H_2_O_2_, HCl, Co­(OAc)_2_·4H_2_O, CuCl_2_·2H_2_O, 1,8-diazabicyclo[5.4.0]­undec-7-ene (DBU), *n*-pentanol, dichloromethane (DCM), dimethylformamide (DMF), ethanol (99.5%), methanol (99.5%), ammonia (25%), and acetone (99.5%) were purchased and used as received. Ultrapure water (UPW) (resistivity [18 MΩ cm]) was produced by a Millipore System. 3-(Hexyloxy)­phthalonitrile was prepared as previously reported.^55^

### 2.2 Materials and Methods

FT-IR spectra were recorded using a Bruker IFS66 FT-IR spectrometer equipped with MCT and DSTG detectors and an Attenuated Total Reflectance (ATR) crystal of germanium in the spectral ranges of 4000 and 600 cm^–1^. An Agilent Carry 60 UV–vis spectrophotometer was used to study the characterization of **CoPc­(OHex)**_**4**_ or **CuPc­(OHex)**_**4**_ and the association of MPcs with rGO. Pure solvents were always used as a reference to fix the baseline. X-ray photoelectron spectroscopy (XPS) measurements were performed with a K Alpha spectrometer from Thermo-Fisher, using a monochromated X-ray Source (Al Kα, 1486.6 eV). For all measurements, a spot size of 400 μm was employed. The hemispherical analyzer was operated in CAE (Constant Analyzer Energy) mode, with a pass energy of 200 eV and a step of 1 eV for the acquisition of survey spectra and a pass energy of 50 eV and a step of 0.1 eV for the acquisition of high-resolution spectra. The spectra obtained were processed using the software provided by Thermo Fisher. A Shirley-type background subtraction was used. The electrochemical measurements were carried with Methrom potensiostat AUTOLAB PGSTAT 100 controlled by NOVA software. Screen printed electrodes were supplied from Dropsens company ref DRC-C110. No uncommon hazards are noted for these works.

### 2.3 Synthesis

#### 2.3.1 Synthesis of Graphene Oxide

Synthesis of graphene oxide was carried out using the modified Hummers method as follows:^54^ H_2_SO_4_ (90 mL, 98%) was added to a volumetric flask containing a mixture of graphite (2.0 g) and NaNO_3_ (2.0 g) at 0 °C with vigorous stirring. After 30 min, KMnO_4_ (10.0 g) was added to the reaction mixture in portions, and the temperature was increased in a controlled manner to 50 °C. After stirring for 2 h, the reaction mixture was cooled down to room temperature, and deionized water (200 mL) and H_2_O_2_ (12.0 mL, 35%) were slowly added to the suspension. The resulting yellow–brown reaction mixture was then washed with HCl (300 mL, 10%). Further washing with concentrated HCl (200 mL, 37%) and with deionized water several times yielded the graphene oxide in a gel-like form. Finally, after freeze-drying for 24 h, graphene oxide was obtained as a black powder.

#### 2.3.2 Synthesis of Reduced Graphene Oxide (**rGO**)

Finely ground graphene oxide (50 mg) and DMF (100 mL) were placed in a flask for ultrasonication for 1 h. The resultant dispersion underwent centrifugation at 1000 rpm for 15 min to remove undissolved GO. Subsequently, the supernatant was collected and stirred in an oil bath at 160 °C for 2 h. The resulting pitch-black mixture was centrifuged at 10,000 rpm, and the precipitated pellets were collected. The pellet was further washed with distilled water, ethanol, and acetone with centrifugation for 30 min to remove any remaining moisture. Finally, the obtained **rGO** was dried at room temperature. Yield 43% (21 mg).

#### 2.3.3 Synthesis of **CoPc­(OHex)**_**4**_

A mixture of 3-(hexyloxy)­phthalonitrile (1 g, 4.38 mmol), Co­(OAc)_2_(H_2_O)_2_ (0.545 g, 2.19 mmol), and DBU (2 mL) in *n*-pentanol (8 mL) was refluxed overnight under N_2_. The dark-colored solution was cooled to room temperature and then poured into water, and the resulting precipitate was filtered. The crude product was purified by column chromatography on silica gel using dichloromethane/ethanol (100/1) as the eluent. Yield: 8% (80 mg). MALDI-TOF-MS (matrix DHB): *m*/*z* 972.057 [M]^+^, calculated for C_56_H_64_CoN_8_O_4_. FT-IR (ν cm^–1^) 2965, 2928, 2861, 1601, 1498, 1461, 1338, 1266, 1106, 1051, 898, 810, 795, 733. UV–vis (CHCl_3_): λ_max_, nm (log ε) = 695 (5.3), 627 (4.6), 310 (4.8).

#### 2.3.4 Synthesis of **CuPc­(OHex)**_**4**_

A mixture of 3-(hexyloxy)­phthalonitrile (1 g, 4.38 mmol), CuCl_2_(H_2_O)_2_ (0.31 g, 2.2 mmol), and DBU (2 mL) in *n*-pentanol (10 mL) was refluxed overnight under N_2_. The dark-colored solution was cooled to room temperature and then poured into water, and the resulting precipitate was filtered. The crude product was purified by several column chromatographies on silica gel using dichloromethane/ethanol (100/1) as the eluent. Yield: 8% (80 mg). MALDI-TOF-MS (matrix DHB): *m*/*z* 976.187 [M]^+^, calculated for C_56_H_64_CuN_8_O_4_. FT-IR (ν cm^–1^) 2975, 2930, 2853, 1603, 1498, 1465 1338, 1278, 1090, 1063, 798, 743. UV–vis (CHCl_3_): λ_max_, nm (log ε) = 708 (4.8), 677 (3.01), 635 (4.1), 317 (4.2).

#### 2.3.5 Preparation of Nanocomposites

The nanocomposites were prepared by dissolving **CoPc­(OHex)**_**4**_ or **CuPc­(OHex)**_**4**_ (1.0 mg) in chloroform (2 mL), and then **rGO** (1.0 mg) was added. The resulting solution was sonicated for 1 h. After the completion, the mixture was placed on a magnetic stirrer for 24 h at room temperature. The precipitated **MPc/rGO** nanohybrid composite was collected using centrifugation at 5000 rpm for 30 min from the supernatant and then washed with water. The synthesized nanocomposites were then kept at room temperature prior to the conductometric studies.

### 2.4 Conductometric Measurements

#### 2.4.1 Description of the Method

The conductometric detection of VOCs was accomplished by applying a specified amplitude with a sinusoidal voltage of 10 mV peak to peak at 0 V to each pair of interdigitated electrodes while using an optimum 10 kHz frequency generated by a “VigiZMeter” conductometer (Covarians (91190 Gif-sur-Yvette, France)). These parameters were chosen to ensure reduced faradaic activity, double-layer charging, and polarization of concentration on the electrode’s surface. The detection was carried out in the headspace above the aqueous phase. Printed circuit technology electrodes were used for sensor fabrication. The electrodes were made of copper (approximately 42 μm) covered with a layer of nickel (3 to 6 μm) and then with a layer of gold (50 to 120 nm). The interdigitated gold electrodes, deposited on a PCB support, have widths of 100 μm and interelectrode distances of 100 μm. The diameter of each sensor (one pair of interdigitated electrodes) was 6 mm. On each chip, there was a working sensor and a reference sensor.

#### 2.4.2 VOC Gaseous Phase Preparation

Gaseous ammonia samples were collected through the headspace above known concentrations of aqueous solutions ranging from 0 to 100 v/v %. At 25 °C, Henry’s law was used to calculate the concentrations of the analyte’s gaseous phase above the aqueous phase from Sander’s equation^56^

1 $$K_{H}^{o}=C_{a}/P_{g}$$

where $K_{H}^{o}$ is Henry’s constant for standard conditions, *C*_a_ is the aqueous phase concentration of the analyte, and *P*_g_ is the partial pressure of the analyte in the gas phase. Henry’s law constants for the analytes considered in these studies are acetone, 20 M/atm; ethanol, 190 M/atm; methanol, 220 × 10^2^ M/atm; and ammonia, 62 M/atm. The equilibrium concentrations of ammonia in the aqueous phase are reported in Table S1, and the equilibrium concentrations of other gases in the gaseous phase as per Henry’s law constants are reported in Table S2, as presented in the literature.^57,58^

## 3 Results and Discussion

### 3.1 Preparation of the **MPc/rGO** Nanocomposites

First, **CoPc­(OHex)**_**4**_ and **CuPc­(OHex)**_**4**_ were synthesized via the cyclotetramerization reaction of 3-(hexyloxy)­phthalonitrile with Co­(OAc)_2_·4H_2_O or CuCl_2_·2H_2_O, respectively, in pentanol in the presence of DBU as a base (Figure 1A). Yields were reproducibly similar, in the 8–10% range. The complete characterization of both Pcs was performed by FT-IR, UV–vis, and MS-MALDI techniques (Figures S1–S6).

**Figure 1:**
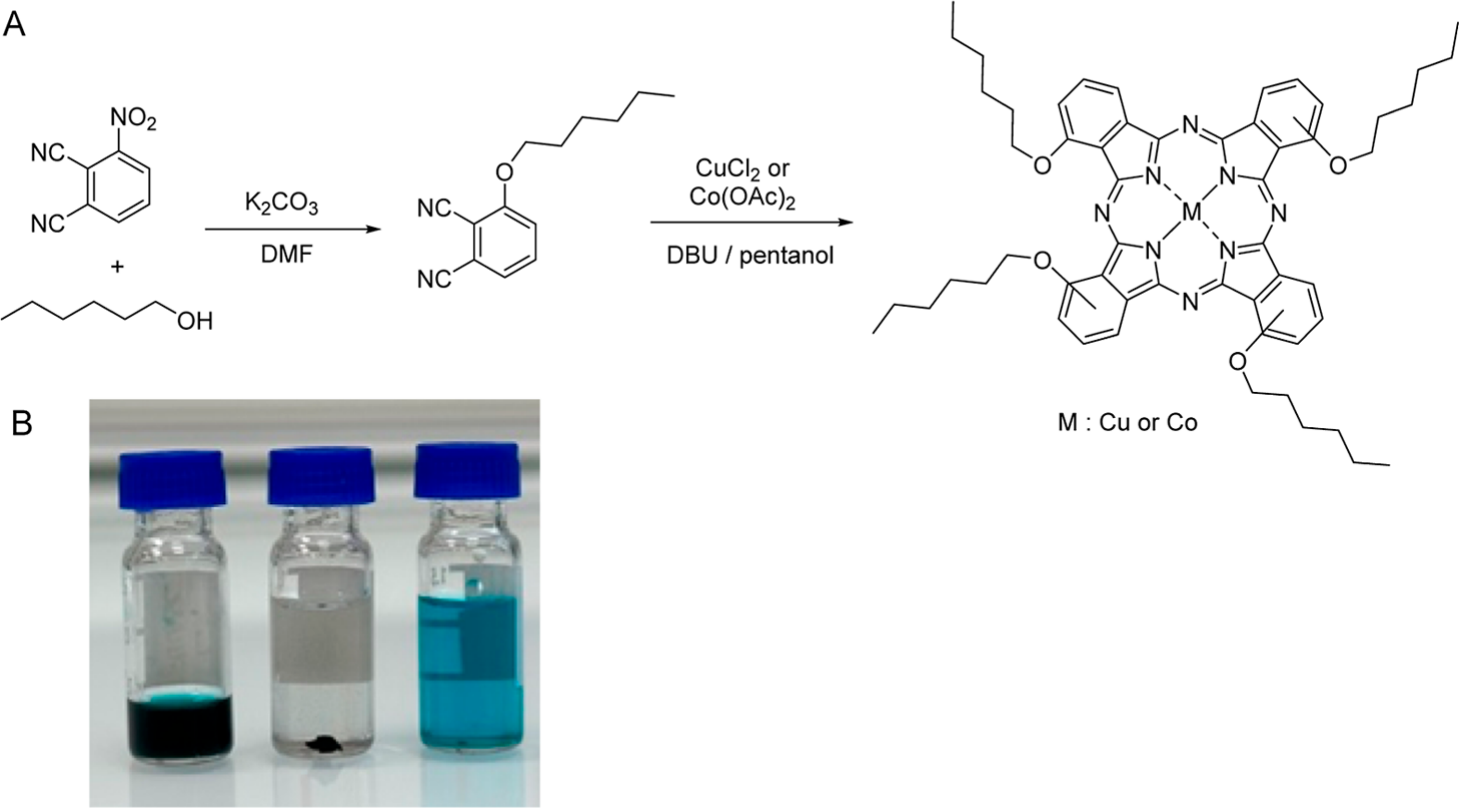
(A) Synthesis of **CoPc­(OHex)**_**4**_ and **CuPc­(OHex)**_**4**_. (B) Left: solution of **CuPc­(OHex)**_**4**_ in chloroform. Middle: **rGO** in chloroform. Right: **CuPc/rGO** nanocomposite dispersed in chloroform (obtained by adding solid **rGO** to a chloroform solution of **CuPc­(OHex)**_**4**_). **rGO** was synthesized by the chemical reduction of graphene oxide prepared according to the modified Hummers method.^54^ The reduction efficiency was analyzed by determining the amount of remaining oxygen during the reduction process by XPS (Figure S7). MPc-rGO nanocomposites were prepared by dissolving the MPc in chloroform and then adding solid **rGO** to the solution. The presence of the **MPc­(OHex)**_**4**_ readily allows the formation of the nanocomposites and their suspension (B). The **MPc/rGO** nanocomposites were very well dispersed in chloroform, thanks to the adsorption of MPc on the graphene structure via π–π interactions. The purification was performed by centrifugation and washing with distilled water to remove the nonadsorbed MPc and **rGO**.

### 3.2 Structural Characterization of the **MPc/rGO** Nanocomposites

The association of MPcs and rGO was studied by UV–vis, FT-IR, and XPS spectroscopies, as well as electrochemistry.

#### 3.2.1 UV–Vis Spectroscopy

The UV–vis spectra of both Pcs in solution show the expected characteristic peaks.^59^ The characteristic single Q-band was observed with λ_max_ values of 695 nm for **CoPc­(OHex)**_**4**_ and 708 nm for **CuPc­(OHex)**_**4**_, indicating that they are fully monomerized in chloroform at micromolar concentration ranges (Figures S3 and S6). The B-band absorptions of **CoPc­(OHex)**_**4**_ and **CuPc­(OHex)**_**4**_ were observed at 310 and 315 nm, respectively. For each nanocomposite, the shape of the Q bands was broadened due to the association of **rGO**, with a small bathochromic shift of 7 nm for **CoPc/rGO** and 2 nm for **CuPc/rGO** (Figure 2). In addition, for the **CuPc/rGO** nanocomposite, the shoulder at 677 nm was broadened and shifted to 681 nm as a result of the association of the MPcs with **rGO** (Figure 2). These shifts can be the result of the reduction in the HOMO–LUMO energy gap of the Pc macrocycle due to the π–π interactions between the Pc cores and **rGO**.^25c,60^ Meanwhile, the B bands of the **CoPc­(OHex)**_**4**_ and **CuPc­(OHex)**_**4**_ were red-shifted to 330 and 351 nm, respectively, and a new band at around 310 nm was observed after the association of MPcs with **rGO** (Figure 2). The new band at around 310 nm is usually ascribed to the π-plasmon of the graphitic structure.^61^ The band gap of all materials was calculated from UV–visible spectroscopy using Tauc plot analysis by plotting (*Ah*ν)^2^ for a direct band gap versus photon energy (*h*ν)^62^ (Figure S8), and data are summarized in Table 1. The band gap values are in the same order for **rGO**, **CoPc­(OHex)**_**4**_, and **CuPc­(OHex)**_**4**_: 3.32, 3.46, and 3.32 eV, respectively. However, in the case of the nanocomposites, a decrease in the band gap to 2.88 eV is observed for **CuPc/GO**, which highlights energy transfer and improved electronic properties.

**Figure 2:**
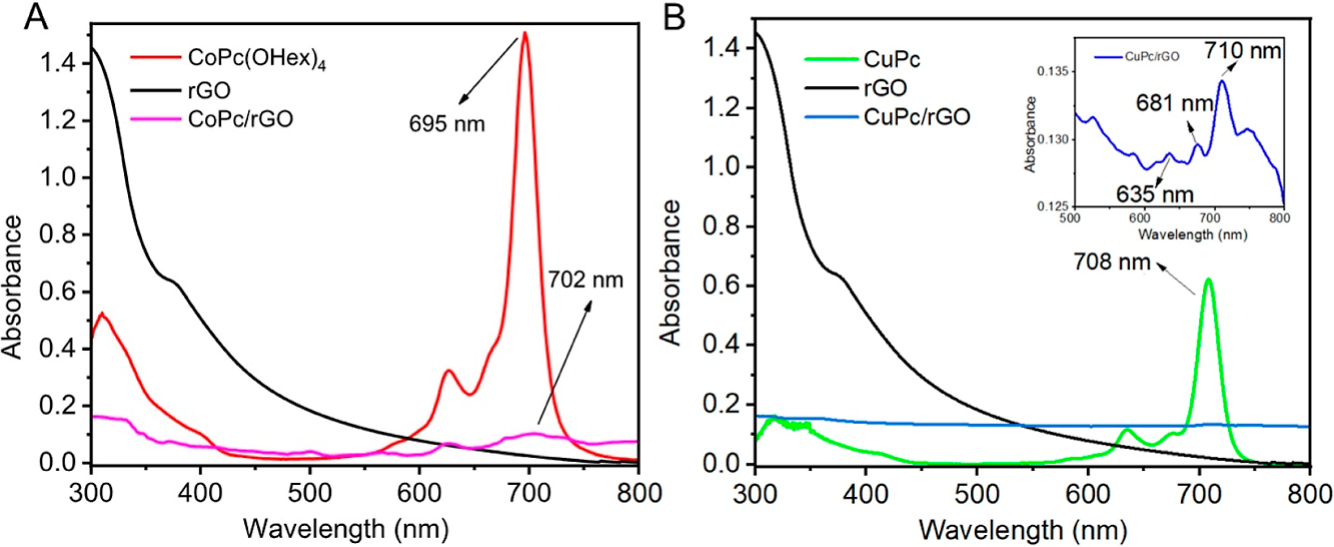
UV–vis spectra. (A) **CoPc­(OHex)**_**4**_ (10 μM in CHCl_3_), **rGO** (1.0 mg/mL in DMF), and **CoPc/rGO** (0.5 mg/mL **CoPc­(OHex)**_**4**_ and 0.5 mg/mL **rGO** in CHCl_3_). (B) **CuPc­(OHex)**_**4**_ (10 μM in CHCl_3_), **rGO** (1.0 mg/mL in DMF), and **CuPc/rGO** (0.5 mg/mL **CuPc­(OHex)**_**4**_ and 0.5 mg/mL **rGO** in CHCl_3_). Inset: plot of extended absorbance (0.10 to 0.16) vs wavelength of the **CuPc/rGO** nanocomposite.

**Table 1:** Characterization of the Nanocomposites and Each Isolated Component

				XPS		CV and ESI		
	UV–vis (nm)		*E*_a_ (eV)	C1_S_ (eV)	M2p^3^ (ev)	Ep_a_ (V)	Δ*E*_p_ (V)	*R*_ct_ (Ω)
**rGO**	298		3.32	284.51		–0.028	0.313	703
**CoPc(OHex)**_**4**_	310	695	3.46	285.22	780.92	–0.182	0.338	566
**CuPc(OHex)**_**4**_	315	708	3.32	285.22	935.31	–0.098	0.463	921
**CoPc/rGO**	330	702	3.49	284.58	780.92	–0.131	0.259	239
**CuPc/rGO**	351	710	2.88	284.82	933.54	–0.03	0.262	286

#### 3.2.2 FT-IR Characterization

FT-IR analysis was performed to evaluate the association between MPcs and **rGO** (Figure 3). For the spectrum of **rGO**, the band at around 1636 cm^–1^ and 1145 cm^–1^ can be attributed to the C=C stretching vibrations and remaining –C–O stretching vibrations after the reduction. The FT-IR spectra of **CoPc­(OHex)**_**4**_ and **CuPc­(OHex)**_**4**_ showed the characteristic peaks of Pc derivatives. The aliphatic −C–H stretches and the −C–O stretching for the non-peripheral substituent were identified at around 2930 cm^–1^, 2864 cm^–1^, and 1117 cm^–1^, respectively. The peaks at 1597 cm^–1^ and 1344 cm^–1^ were attributed to the benzene ring stretching and pyrrole ring stretching, respectively.^60^ After the formation of the nanocomposites, the peaks corresponding to MPcs and **rGO** alone at around 1600 cm^–1^ were observed with small shifts.

**Figure 3:**
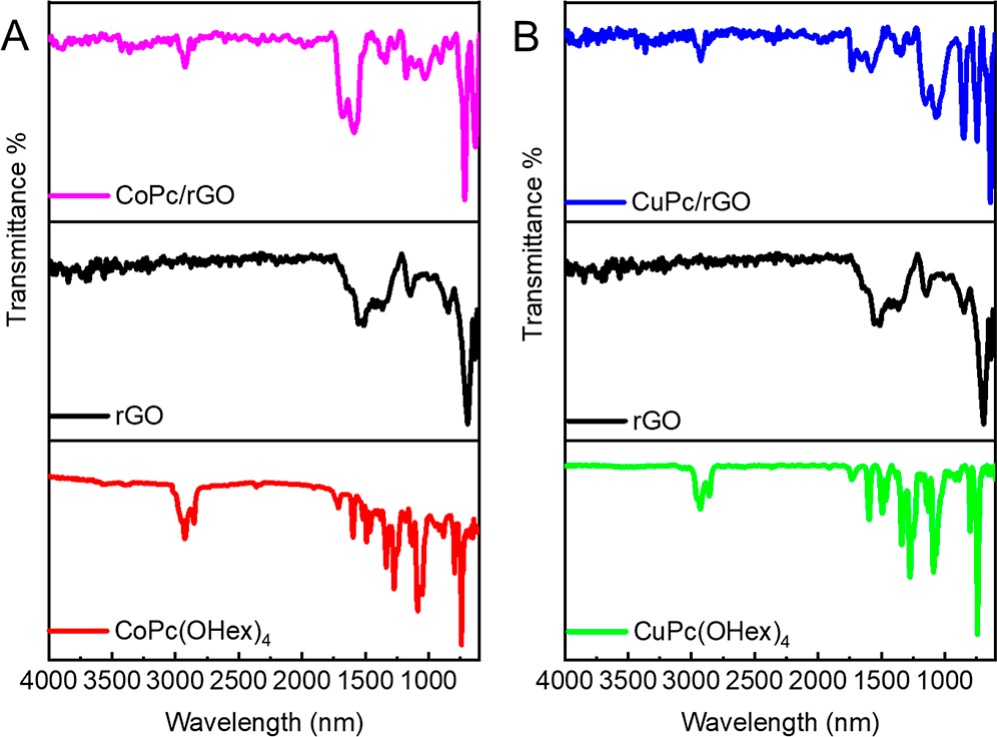
FT-IR spectra of (A) **CoPc­(OHex)**_**4**_, **rGO**, and **CoPc/rGO** and (B) **CuPc­(OHex)**_**4**_, **rGO**, and **CuPc/rGO**.

#### 3.2.3 X-ray photoelectron spectroscopy

XPS characterization is used to study the electronic properties of each material (the MPcs alone, **rGO**, and the nanocomposites). An XPS survey was performed 4 times, and then the deconvolution of each element C, N, O, Cu, and Co was performed for each compound. From XPS, we can learn about the modification of the graphene Fermi level from the location C 1s position. The ability of the groups to donate or remove electrons causes the binding energy peak of C=C to shift up or down due to charge transfer. In the same way, the association of **rGO** could affect the energy levels of the metal ions in macrocycles. By comparing their energy level before and after interaction,^63^ we can clearly determine if the metal ions are also in interaction with **rGO** and the effect of **rGO** on their charge. Figure 4 presents the spectra obtained with **CuPc/rGO** and **CoPc/rGO** in comparison with **rGO**, **CuPc­(OHex)**_**4**_, and **CoPc­(OHex)**_**4**_.

**Figure 4:**
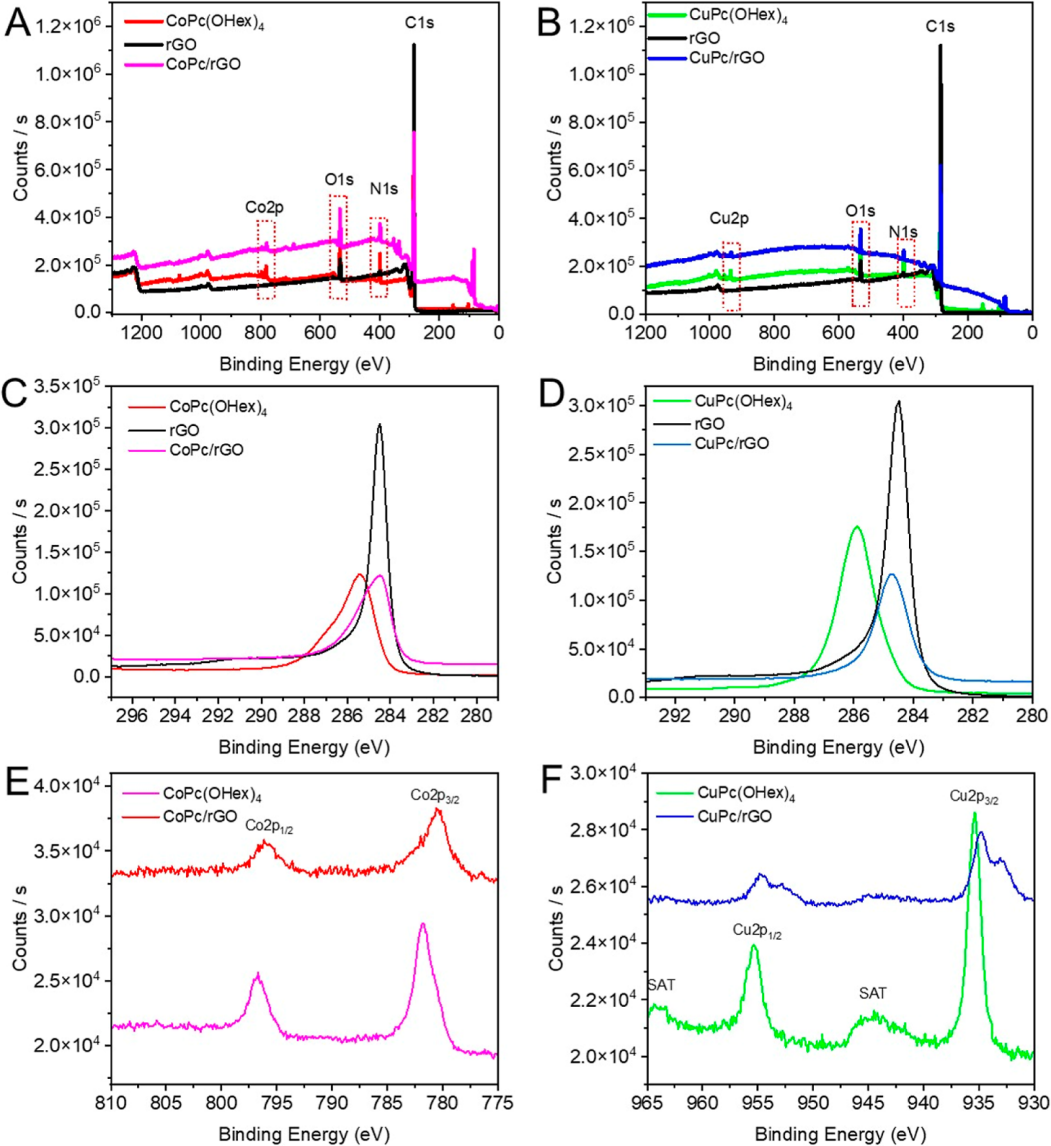
XPS spectra. (A) Survey spectra of **CoPc­(OHex)**_**4**_, **rGO**, and **CoPc/rGO**; (B) survey spectra of **CuPc­(OHex)**_**4**_, **rGO**, and **CuPc/rGO**; (C) high-resolution C 1s for **CoPc­(OHex)**_**4**_, **rGO**, and **CoPc/rGO**; (D) high-resolution C 1s for **CuPc­(OHex)**_**4**_, **rGO**, and **CuPc/rGO**; (E) high-resolution Co 2p for **CoPc­(OHex)**_**4**_, **rGO**, and **CoPc/rGO**; and (F) high-resolution Cu 2p for **CuPc­(OHex)**_**4**_, **rGO**, and **CuPc/rGO**.

The nanocomposites show the shift of the position of the C 1s, Cu 2p^3^, and Co 2p^3^ peaks compared to MPcs (Table 1). This shift can be related to the effect of the association of MPc with **rGO**. It was demonstrated that depending on the electron-donating or withdrawing groups, charge transfer leads to upshifts or downshifts in the C=C peak.^64^ A downshift of the C 1s peak in eV was observed as −0.2 and −0.28 eV for, respectively, **CoPc**/**rGO** and **CuPc**/**rGO**. This modification is consistent with charge transfer between the aromatic molecules and graphene sheets, where **CuPc­(OHex)**_**4**_ and **CoPc­(OHex)**_**4**_ have withdrawing effects.

In the case of the metal ions, the survey shows a 2p peak of metal at 780.92 and 935.31 for, respectively, **CoPc­(OHex)**_**4**_ and **CuPc­(OHex)**_**4**_. For the nanocomposites, a small variation was observed in Co 2p energy (Table 1). In the case of **CuPc/rGO**, a downshift of −1.77 eV was observed, demonstrating energy transfer between metal ions and **rGO**. The deconvolution of the Cu 2p and Co 2p in the MPcs and in the nanocomposites shows the presence of two peaks corresponding to M2p_1/2_ and M2p_3/2_, respectively, demonstrating Co­(II) and Cu­(II) oxidation states of metal^65^ (Figure 4E,F). Multiple splitting satellites are observed, showing that the macrocycle is planar and, indeed, the presence of the functional groups. Deconvolution of **CuPc/rGO** spectra (Figure 4F) shows a splitting of Cu 2p_1/2_ and Cu 2p_3/2_ with the new peaks (Table S3). This new band could be related to possible interactions of **CuPc­(OHex)**_**4**_ with **rGO** in the composite: one with the functional groups and the other with macrocycles involving the metal ions. In the case of **CoPc/rGO**, a downshift of Co 2p is observed for Co 2p_1/2_ and Co 2p_3/2_ without splitting, demonstrating that the interaction of **rGO** with the macrocycle is dominating.

#### 3.2.4 Electrochemical Analysis

To study the surface properties and electrochemical behavior of **rGO** and of the two nanocomposites, cyclic voltammetry (CV) and electrochemical impedance spectroscopy (EIS) were performed, using a redox marker in solution, [Fe­(CN)_6_]^3–/4–^, which is an inner-sphere complex used to underline the surface properties of the composites and electron transfer ability.^66^ CV curves of the electrodes modified with the nanocomposites in comparison with the product alone are presented in Figure 5, and they show an increase in the current peak and decrease in the peak potential for both oxidation and reduction for nanocomposites **CoPc/rGO** and **CuPc/rGO**. The shift is due to the charge transfer induced by the interaction between **rGO** and the MPcs. The redox process for the [Fe­(CN)_6_]^3–/4–^ marker becomes easier when the nanocomposites are formed due to charge transfer between MPcs and **rGO**. These observations confirm the results observed previously in UV–vis spectroscopy and XPS. In addition, an increase in the current was observed for both nanocomposites, which confirms the interaction between the two compounds and **rGO**, and then an increase of surface area. The surface area was calculated using the Randles–Sevcik equation (Table S4) and showed an increase of the electroactive surface area in the case of the nanocomposites compared to **rGO**.

**Figure 5:**
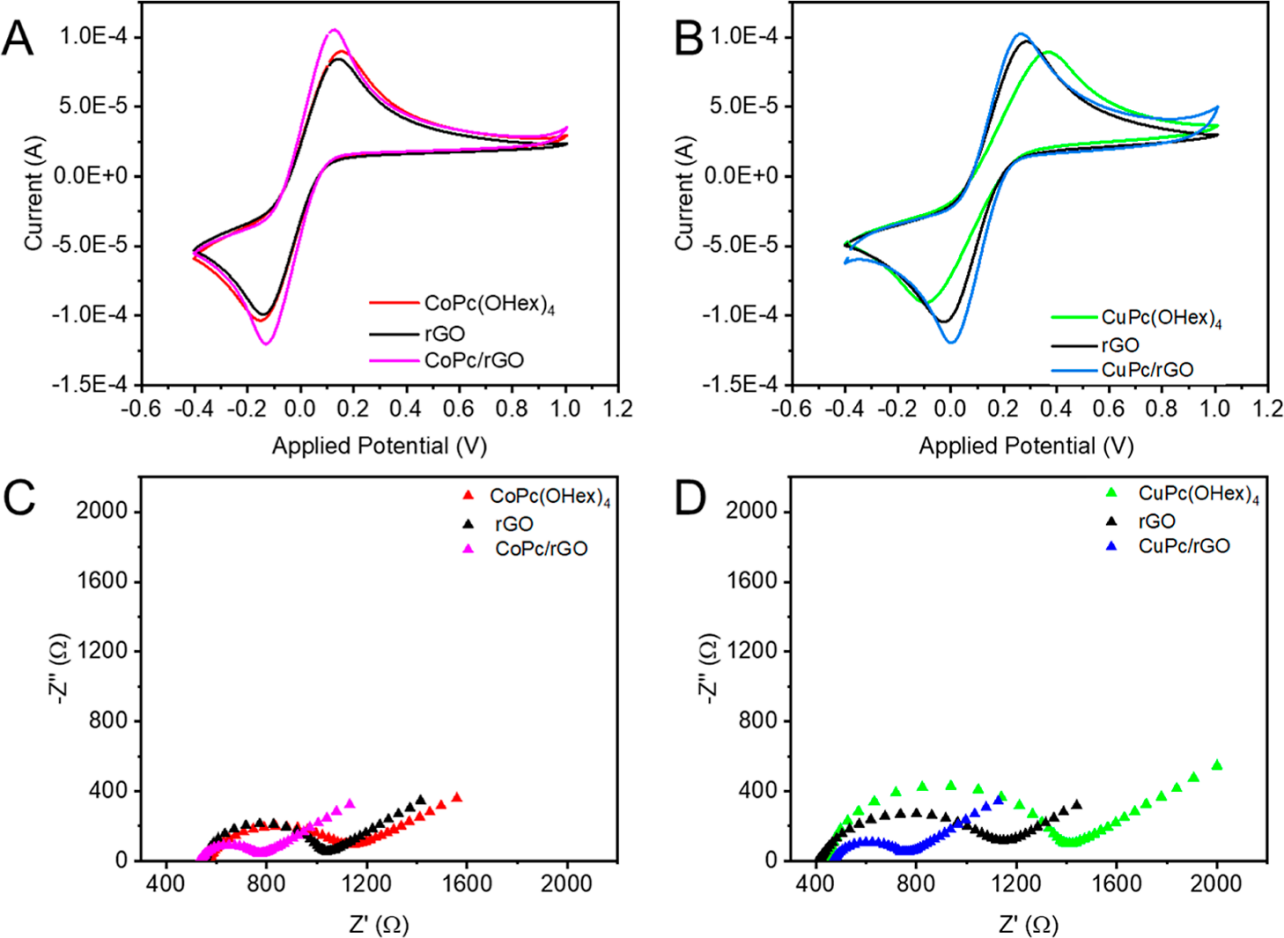
Cyclic voltammograms of SPCE modified with (A) **CoPc­(OHex)**_**4**_, **rGO**, and **CoPc/rGO** and (B) **CuPc­(OHex)**_**4**_, **rGO**, and **CuPc/rGO** in 10 mM K_3_[Fe­(CN)_6_]/K_4_[Fe­(CN)_6_] (1:1) in 0.1 M KCl with a scan rate of 0.05 V s^–1^. Nyquist plots (−Z″ vs Z′) obtained from impedance measurements in the presence of solution [Fe­(CN)_6_]^3–/4–^ redox at 100 kHz to a 0.1 Hz frequency range and a DC potential of 10 mV for SPCE modified with (C) **CoPc­(OHex)**_**4**_, **rGO**, and **CoPc/rGO** and (D) **CuPc­(OHex)**_**4**_, **rGO**, and **CuPc/rGO**.

EIS was also performed to determine the electrical properties and to highlight the charge transfer between the MPcs and **rGO**. The Nyquist curves were plotted with **rGO**, **CoPc/rGO**, and **CuPc/rGO** (Figure 5C,D) and showed a semicircle for both the modified surfaces. A decrease of impedance was observed for the nanocomposites compared to **rGO** and MPc. The fitting data obtained with the equivalent circuit model (Table S5) showed a decrease of charge transfer resistance after nanocomposite formation, where the *R*_ct_ decreased from 475 Ω for rGO to 286 Ω and 239 Ω for **CuPc/RGO** and **CoPc/rGO**, respectively. These results clearly confirm that the association of **CuPc­(OHex)**_**4**_ and **CoPc­(OHex)**_**4**_ with **rGO** has an electronic effect on **rGO** due to the presence of the aromatic ring of macrocycles and the four electron-donating alkoxy groups, increasing the charge transfer ability.

### 3.3 Gas Detection

The gas sensing performance of the **CuPc/rGO** and **CoPc/rGO** nanocomposites was studied and compared to those of the Pcs alone and of **rGO** toward various VOCs, with the aim of investigating the effect of the Co or Cu metalation of the MPc and of the association with **rGO** on VOCs using the conductometric detection method. Various sensors were prepared on gold interdigitated electrodes by drop casting of **rGo**, **CuPc­(OHex)**_**4**_, **CoPc­(OHex)**_**4**_, **CuPc/rGO**, and **CoPc/rGO** nanocomposites on the surface of the chips, using one working electrode modified with the materials and the second electrode as a reference (Figure 6).

**Figure 6:**
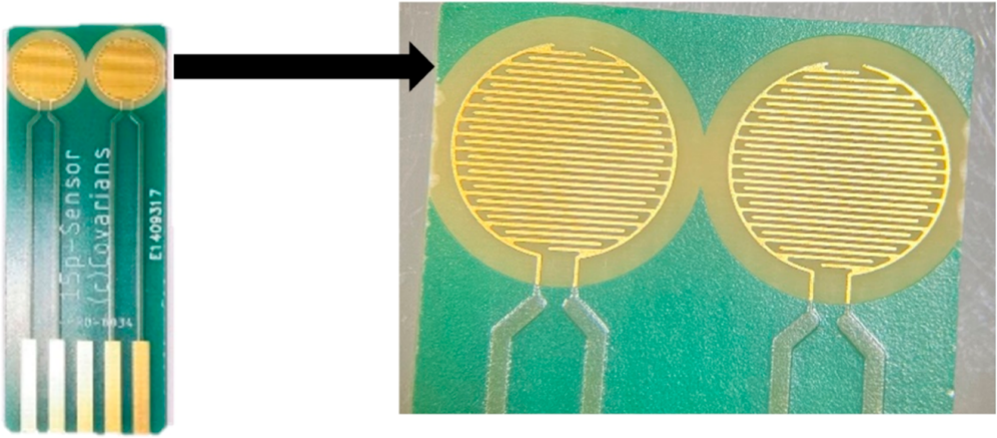
Microconductometric chips fabricated with Interdigitated gold electrodes fabricated by the printed circuit technology: working sensor and reference sensor.

#### 3.3.1 Humidity Detection

First, the effect of humidity on sensor response was tested (Figure 7). The results show that each Pc alone has a very small response to water due to its hydrophobicity. With both nanocomposites, no response was obtained, demonstrating the high hydrophobicity of **rGO** associated with MPcs, likely due to the presence of the alkyl groups enhancing the hydrophobicity of the nanocomposite.

**Figure 7:**
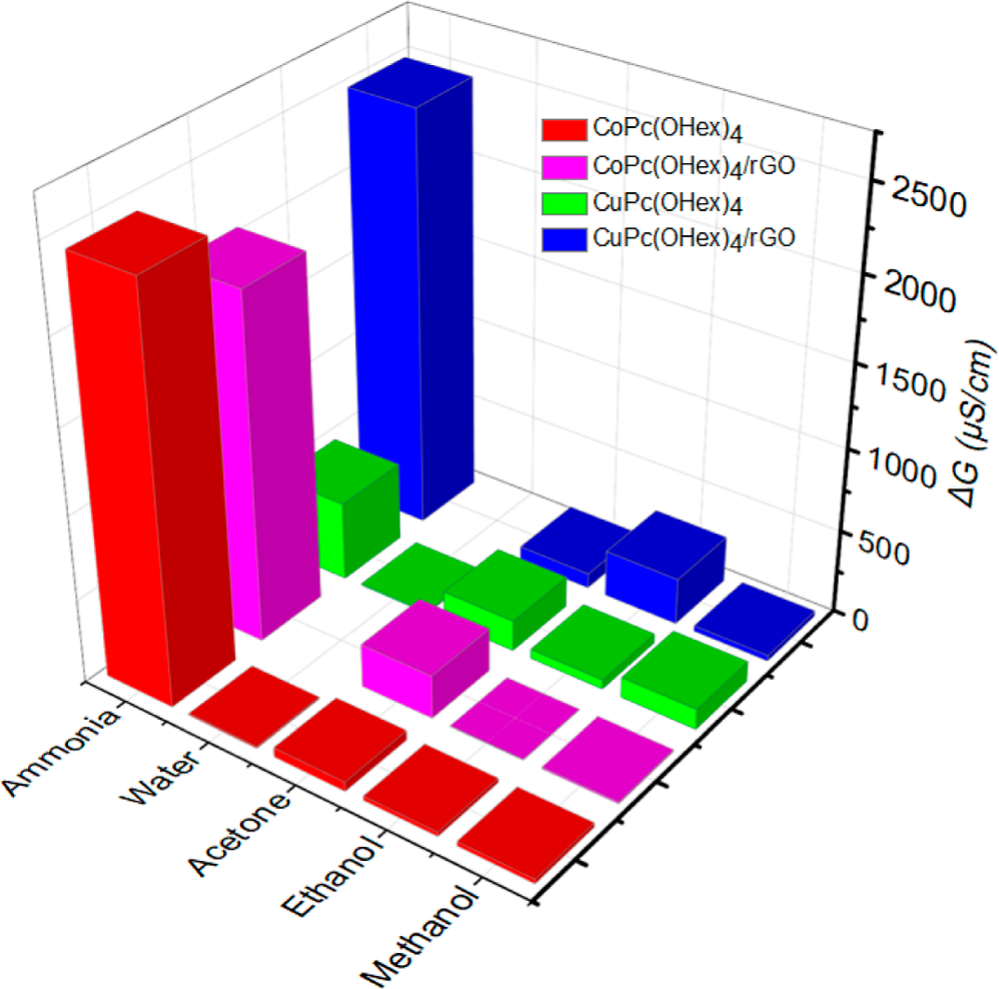
Detection of water and VOCs at high concentration levels in gas-phase (ammonia, 17.3; water, 3; acetone, 56.2; ethanol, 9.01; methanol, 11.2) by the MPcs and the nanocomposites.

#### 3.3.2 VOC Detection

Next, the sensing of several VOCs was tested: acetone, ammonia, ethanol, and methanol (Figure 7). All the sensors show much higher sensitivity to ammonia compared to acetone, ethanol, and methanol. It could be explained by the affinity of ammonia for MPcs and be a result of the more favorable electron donor–acceptor interaction between the MPc and the NH_3_ molecule. Both **MPc/rGO** nanocomposites demonstrated the best response to ammonia compared to the MPcs alone. This can be related to the noncovalent interaction arising from the synergetic effect between MPcs and **rGO**, offering a larger surface area for NH_3_ diffusion and, accordingly, more active sites for adsorption of NH_3_.

The high sensitivity of **CuPc/rGO** and **CoPc/rGO** nanocomposites to ammonia compared to other VOCs can be explained by their strong electron donor character that improves the density of metal ions in MPcs and then the charge transfer to **rGO**, providing an increase in conductivity. This matches previous reports, when the association of ammonia with various unsubstituted MPcs (including **CuPc** and **CoPc**) using X-ray crystallography experiments, energy band calculation, and density functional theory (DFT) calculations was studied and it was shown that 3d_*z* ^2^_ of Cu and Co and 2p_*z*_ of N (NH_3_) are involved in the interaction between metal and nitrogen, providing strong interaction between the metal site and ammonia, leading to electron donating ability and high charge transfer.^23^ Some other studies also demonstrated higher sensitivity **of MPc/rGO** nanocomposites to ammonia compared to other VOCs.^25c,53,67^ In addition, the highest affinity of **CuPc­(OHex)**_**4**_—compared to **CoPc­(OHex)**_**4**_—is explained by a lower distance between Cu and N in the complex.

#### 3.3.3 Sensors of Ammonia Based on **CoPc­(OHex)**_**4**_, **CoPc/rGO**, **CuPc­(OHex)**_**4**_, and **CuPc/rGO**

The sensor responses of **CoPc­(OHex)**_**4**_ and of **CoPc/rGO** sensors were then tested with increasing concentrations of ammonia. It can be concluded from Figure 8 that the conductivity of the sensors increases with the NH_3_ concentration in the gas phase and that the sensors exhibit reversible and stable responses even at the lower concentrations of ammonia. For the highest concentrations of NH_3_, only small differences were observed between **CoPc­(OHex)**_**4**_ alone and the corresponding nanocomposite **CoPc/rGO**; the nanocomposite showed linear variation compared with the Pc alone. This could be related to the high surface area provided by **rGO**.

**Figure 8:**
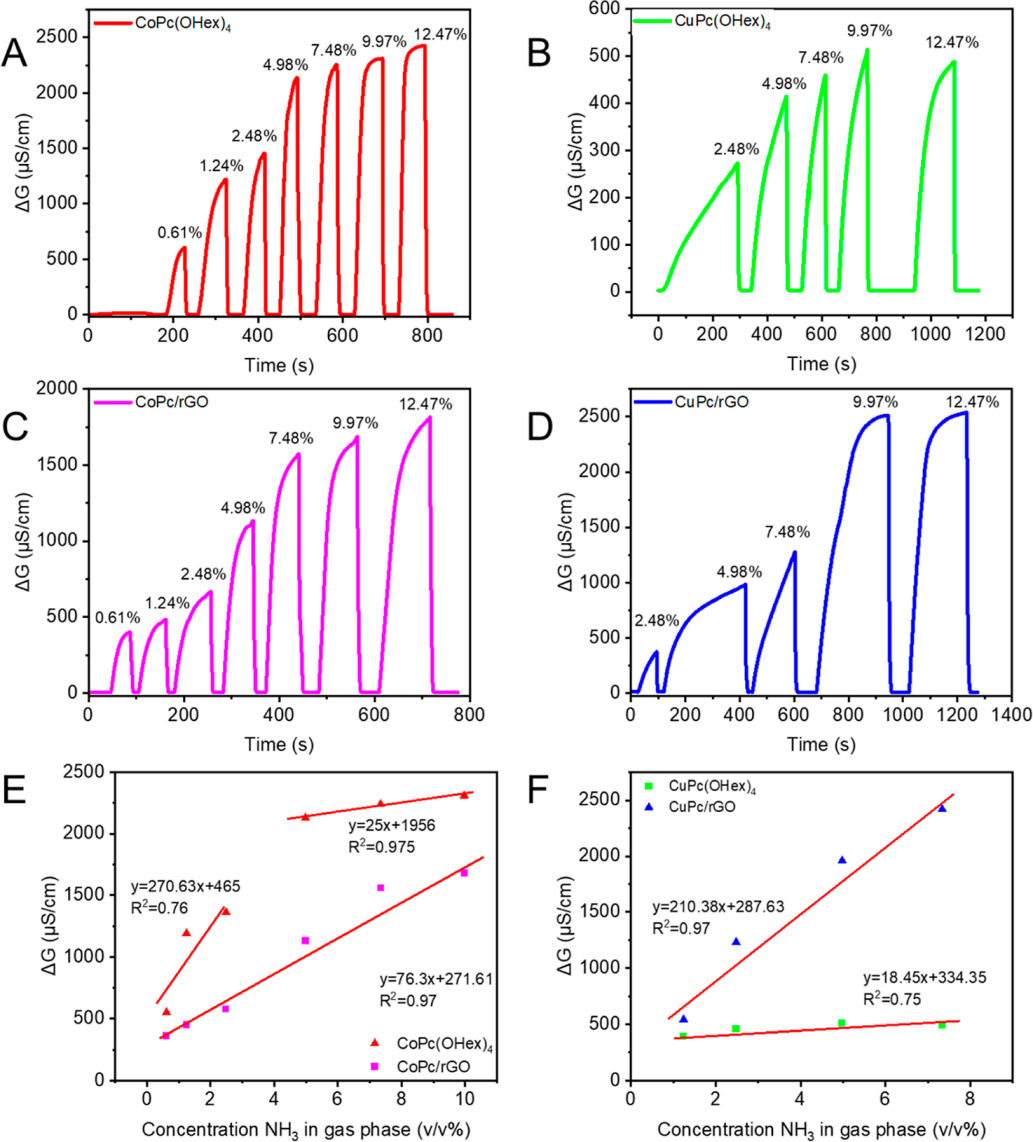
Detection of gas-phase concentration for various concentrations of ammonia with (A) **CoPc­(OHex)**_**4**_, (B) **CuPc­(OHex)**_**4**_, (C) **CoPc/rGO**, and (D) **CoPc/rGO** nanocomposite sensors and calibration curve presenting the maximum value of resistance within the concentration of ammonia in gas phases with (E) **CoPc­(OHex)**_**4**_ and **CoPc/rGO** and (F) **CuPc­(OHex)**_**4**_ and **CuPc/rGO** nanocomposite sensors.

In the case of **CuPc­(OHex)**_**4**_ and **CuPc/rGO**, the nanocomposite **CuPc/rGO** has high sensitivity for ammonia compared to **CuPc­(OHex)**_**4**_: NH_3_ sensing was nearly 5 times higher signal conductivity compared to **CuPc­(OHex)**_**4**_. As the gas sensing is described by the adsorption to and from the surface, this behavior can be the consequence of the grown surface area with the noncovalent interaction of **CuPc­(OHex)**_**4**_ and **rGO**, which also offers more space for diffusion and effusion sites for NH_3._

The sensitivity was also tested with **rGO**, and only a small response was obtained (Figure S9), which agrees with the literature.^68^ This highlights the high effect of MPcs in ammonia sensing, which is related to their association with metal ions of macrocycles, as explained in the previous section.

#### 3.3.4 Analytical Performance

The calibration curves were plotted by measuring the variation of the conductance within the concentration with the four sensors. In the case of **CoPc/rGO** and **CuPc/rGO** (Figure 7E,F), the calibration curves exhibit linear response to NH_3_ with a high coefficient of regression of 0.97, and the slopes give the value of sensitivity. The reproducibility of the ammonia sensor was tested with different concentrations and different sensors, the linearity of the curve was tested, and a standard deviation of 5% was calculated with 3 independent measurements. The detection limit calculated from the slope of the curve and considering the standard deviation and signal-to-noise ratio of 3 is calculated (Table 2).

**Table 2:** Comparison of the Analytical Performances of the Sensors

	sensitivity (μS/cm)/v/v %	LOD (ppm)	response time (s)	recovery time (s)
**rGO**			118.73	1.25
**CoPc(OHex)**_**4**_			32.58	5.88
**CuPc(OHex)**_**4**_	25.6	5.7	247.43	14.3
**CoPc/rGO**	112	1	26.73	4.9
**CuPc/rGO**	309	0.4	68.06	2.11

The sensor formed with **CoPc­(OHex)**_**4**_ showed random response with concentration, where high variation with low concentration and then saturation can be seen. This can probably be due to two association modes of ammonia with the Pc: with the macrocycle and with the non-peripheral substituents. In the case of the nanocomposites, the **CoPc­(OHex)**_**4**_ and **CuPc­(OHex)**_**4**_ stack on **rGO**, leading first to an increase of surface area and high accessibility of the ammonia for coordination to the macrocycle. This improvement of charge transfer between MPc and **rGO** by the donating effect, as we show in XPS and band gap measurement, favored the absorption of ammonia. We can highlight that hexyloxy substituents have a positive effect on the gas sensitivity of ammonia. Other literature data also showed a positive effect of oxygen-containing substituents on Pcs for ammonia sensing capacity. CoPc modified with carboxylic groups and conjugated to **rGO** showed higher sensitivity to ammonia compared to Pc alone and **rGO**. Other CoPcs substituted with various oxygen-containing moieties, either flexible or rigid, and associated with **rGO**, are also demonstrated to favor ammonia detection, which was explained by a positive effect of these substituents.^25c^ The tetra-isopentyloxy-substituted phthalocyanine CuPc-based rGO nanocomposite also showed good sensitivity to ammonia for the same reasons.^67^ The data obtained from various NH_3_ sensors based on Pc/rGO nanocomposites are summarized in Table S6, evidencing the best results of our materials.

The response time (*t*_Res_) and recovery time (*t*_Rect_) are commonly used to describe the temporal characteristics of the sensors. The response time refers to the duration required for the conductance to reach 90% of its total change upon stimulus application. On the other hand, the recovery time is the duration needed for the conductance to return by 10% of its total change.^69^ As shown in Table 2, introducing **rGO** to the **CoPc­(OHex)**_**4**_ and **CuPc­(OHex)**_**4**_ macrocycles via π–π stacking led to a decrease in both response time and recovery time. This behavior can be attributed to the enhanced charge transfer and the increased surface area arising from the noncovalent interaction between MPcs and **rGO**.^70^

#### 3.3.5 Detection in Real Sample Measurements

The detection in the real sample was performed with commercial ammonia solution, where the concentration given by the provider is 12%. The conductivity of this solution was measured with various sensors: **CuPc­(OHex)**_**4**_, **CuPc/rGO**, **CoPc­(OHex)**_**4**_, **CoPc/rGO**, and **rGO**. The values obtained are, respectively, 278 μS/cm, 390 μS/cm, 628 μS/cm, and 1140 and 1015 μS/cm. The corresponding concentrations were determined from a calibration curve obtained previously, and the headspace (gaseous phase) volume of 10.80 ± 1.44 v/v %, and the equivalent molar concentration of 6.69 ± 0.79 M in the aqueous phase 11.3 ± 1.3% were determined with the recovery calculated at 94%.

## 4 Conclusions

These results demonstrate that the association of hexyloxy-tetrasubstituted **CuPc** and **CoPc** with **rGO** gives nanocomposites with improved electronic properties, where a decrease in band gap is measured and charge transfer is demonstrated. The nanocomposites were employed for VOCs sensing using conductometric measurement by drop casting on the surface of an interdigitated electrode. The sensors formed with our **MPc/rGO** nanocomposites have high sensitivity to ammonia compared to other VOCs. This can be related to the electronic structure of the Pc macrocycle and its affinity for metal ions to coordinate the ammonia. We highlight the positive effect of the nanocomposite with **rGO** in improving the electronic properties and then the ability for ammonia sensing. The sensors formed with composite have very high sensitivity with estimated detection limits at 1 and 0.4 ppm for **CoPc/rGO** and **CuPc/rGO**, respectively. The sensor also presents the ability to detect ammonia in real samples with a recovery as high as 94%.

In summary, this work demonstrates that suitable molecular engineering of phthalocyanines offers a powerful approach to overcoming solubility and electronic compatibility challenges in the fabrication of MPc/rGO nanocomposites. The scalable synthesis method developed here enables the full utilization of graphene sheet properties, leading to an enhanced charge transfer and gas sensing performance. Beyond the specific case of ammonia detection, these findings provide a broader framework for the design of hybrid materials, where tailored inorganic macrocycles are key to tuning interfacial interactions and device performance.

Further works beyond the scope of this study will deal with the investigation of the properties of our novel sensors toward other reducing gases that are not actual VOCs, such as CO and H_2_, to extend the impact of our research to other sensing applications in other contexts.

## Supplementary Material



## Notes

Characterization spectra of the phthalocyanines and of the composites; calibration curves and fitting parameters for the sensing experiments; and literature data for comparison purposes (PDF)
