# Supplementary material for: CoPc/rGO and CuPc/rGO Nanocomposites: Comparative Characterization and Study of Their Gas Sensing Properties
Source: Inorg Chem. 2025 Jun 26;64(27):13740–51. doi: 10.1021/acs.inorgchem.5c01085 (PMC12264944; doi:10.1021/acs.inorgchem.5c01085)
Supplement: Supplementary file 1 [file ic5c01085_si_001.pdf]

Supporting Information for

CoPc/rGO and CuPc/rGO nanocomposites: comparative characterization and study of their gas sensing properties

Çiğdem Yağcı[1,2]†, Oluwatoyin Emmanuel Aina[3]†, Atefeh Emami[4], Amal Ibijbijen[1], Diana Dragoe[1], Haifa Ben Aziza[4], Ümit İşci[5], Abdelhamid Errachid [3], Nicole Jaffrezic Renault[6], Fabienne Dumoulin*[4], Hafsa Korri-Youssoufi*[1]*

[1] Université Paris-Saclay, ICMMO, UMR-CNRS, 17 avenue des sciences 91405 Orsay, France

[2] Kocaeli University, Faculty of Education, Umuttepe Campus, 41001 İzmit Kocaeli, Türkiye

[3] Université Claude Bernard Lyon-1, CNRS, ISA-UMR 5280, CNRS, 5 Rue de la Doua 69100 Villeurbanne, France

[4] Acıbadem Mehmet Ali Aydınlar University, Faculty of Engineering and Natural Sciences, Biomedical Engineering Department, 34752 Ataşehir Istanbul, Türkiye

[5] Marmara University Faculty of Technology, Metallurgical and Materials Engineering Department, 34854 Maltepe, Istanbul, Türkiye

[6] University of Franche-Comte, UTINAM Institute, 16 Gray road, 25030 Besancon, France

* Corresponding authors:

fabienne.dumoulin@acibadem.edu.tr

hafsa.korri-youssoufi@universite-paris-saclay.fr

nicole.jaffrezic-renault@univ-fcomte.fr

Table of Contents

Figure S1. MALDI-TOF mass spectrum of **CoPc(OHex)$_4$** …………………………………….. S3

Figure S2. FT-IR spectrum of **CoPc(OHex)$_4$** ………………………………………………. S3

Figure S3. UV–Vis electronic absorption spectra of **CoPc(OHex)$_4$** (chloroform, 1-10 µM). Inset: Plot of absorbance vs. concentration at 695 nm……………………………………… S4

Figure S4. MALDI-TOF mass spectrum of **CuPc(OHex)$_4$** …………………………………….. S4

Figure S5. FT-IR spectrum of **CuPc(OHex)$_4$**……………………………………………….. S5

Figure S6. UV–Vis electronic absorption spectra of **CuPc(OHex)$_4$** (chloroform, 1-10 µM). Inset: Plot of absorbance vs. concentration at 708 nm……………………………………… S5

Figure S7. XPS survey of **rGO** ……………………………………………………… S6

Figure S8. Determination of the band gap of (a) **CoPc(OHex)$_4$**, (b) **CuPc(OHex)$_4$**, (c) **CoPc/rGO**, (d) **CuPc/rGO** (e) **rGO**…………………………………………………… S7

Figure S9. Detection of gas-phase concentration for various concentration of ammonia with **rGO** sensor (a), and calibration curve presenting the maximum value of resistance within the concentration of ammonia in gas phases with rGO (b)…………………………………... S8

Table S1. Equilibrium aqueous phase molar concentrations of analytes…………………… S9

Table S2. Equilibrium gas phase molar concentrations of analytes at 25 ^{o}C per Henry's law constants……………………………………………………………………………… S10

Table S3. XPS Binding energy values of nanocomposites and of their isolated components for 2p$_{1/2}$ and 2p$_{3/2}$ states……………………………………………………………….. S11

Table S4. The electroactive surface of the nanocomposites and of their isolated components……………………………………………………………………………… S11

Table S5. Fitting parameters of EIS data…………………………………………………… S12

Table S6. A comparison of various sensors on NH_3 sensing………………………………... S13

Determination of the electroactive surface area……………………………………………….. S14

References……………………………………………………………………………………... S15

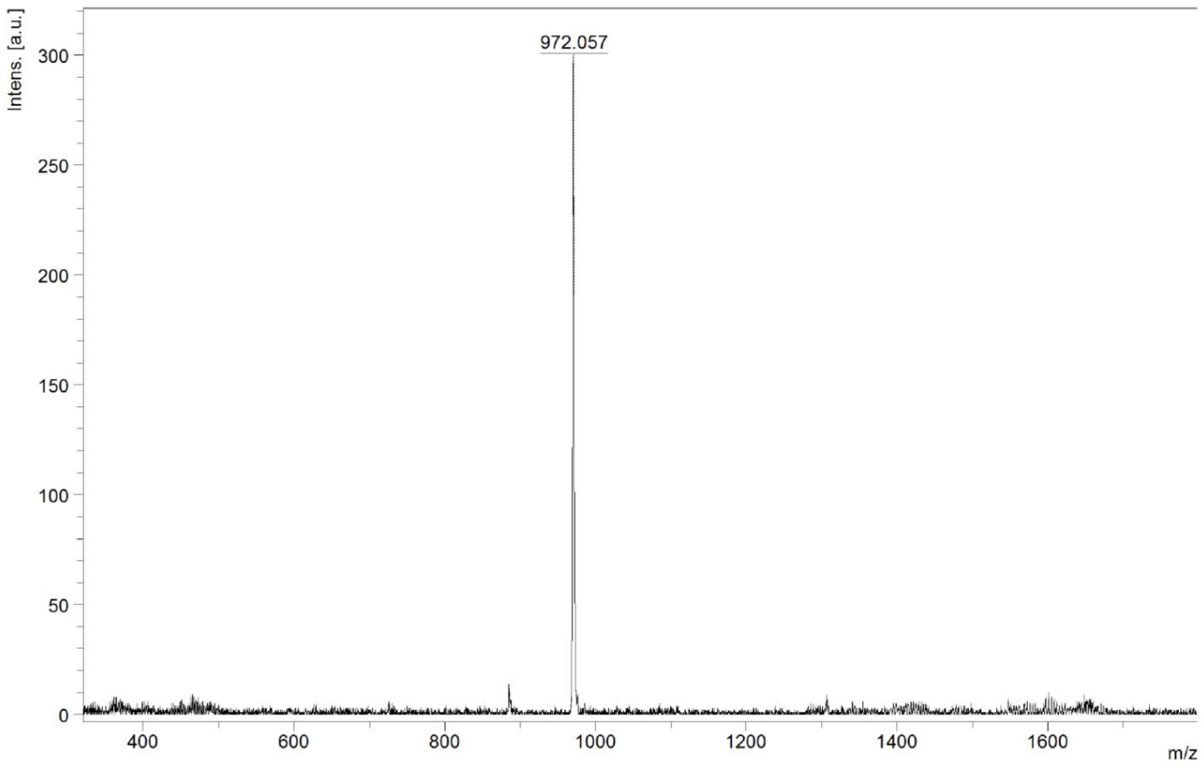

Figure S1. MALDI-TOF mass spectrum of **CoPc(OHex)$_4$** (matrix: DHB).

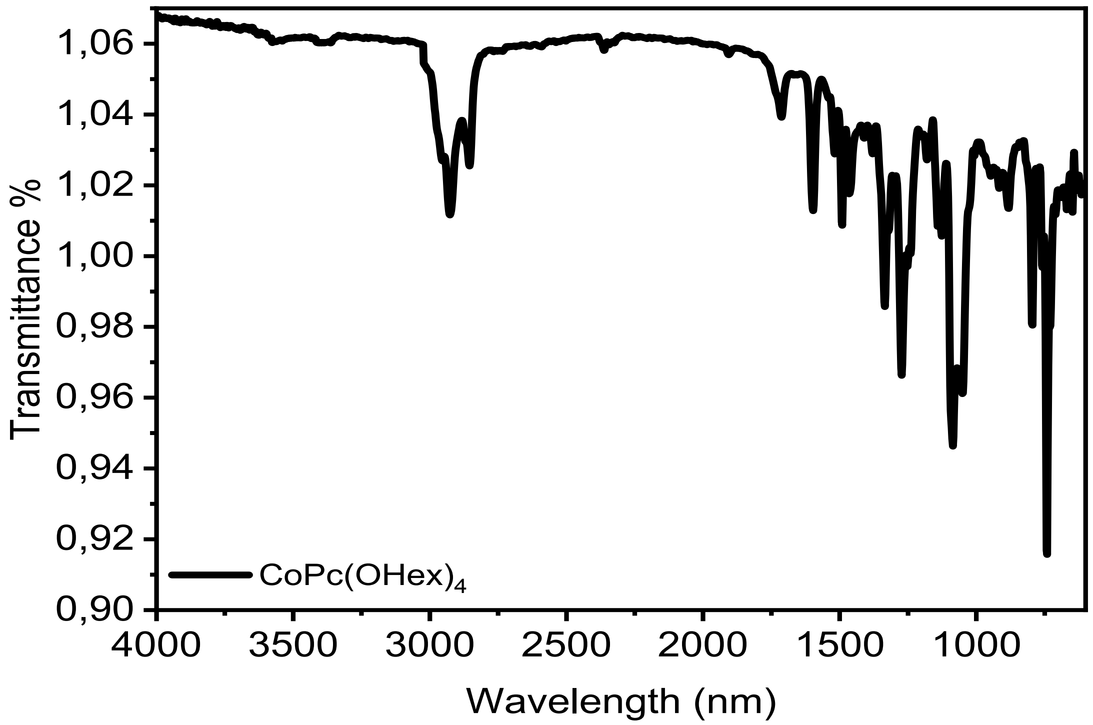

Figure S2. FT-IR spectrum of **CoPc(OHex)$_4$**.

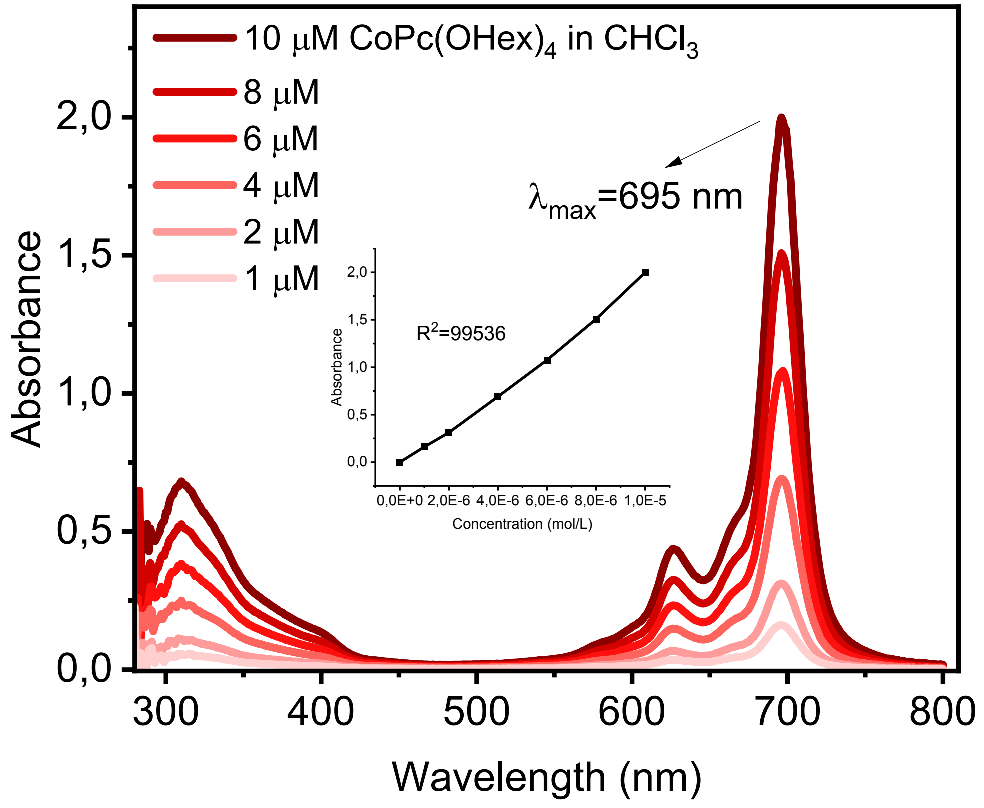

Figure S3. UV–Vis electronic absorption spectra of **$CoPc(OHex)_4$** (chloroform, 1-10 μM). Inset: Plot of absorbance vs. concentration at 695 nm.

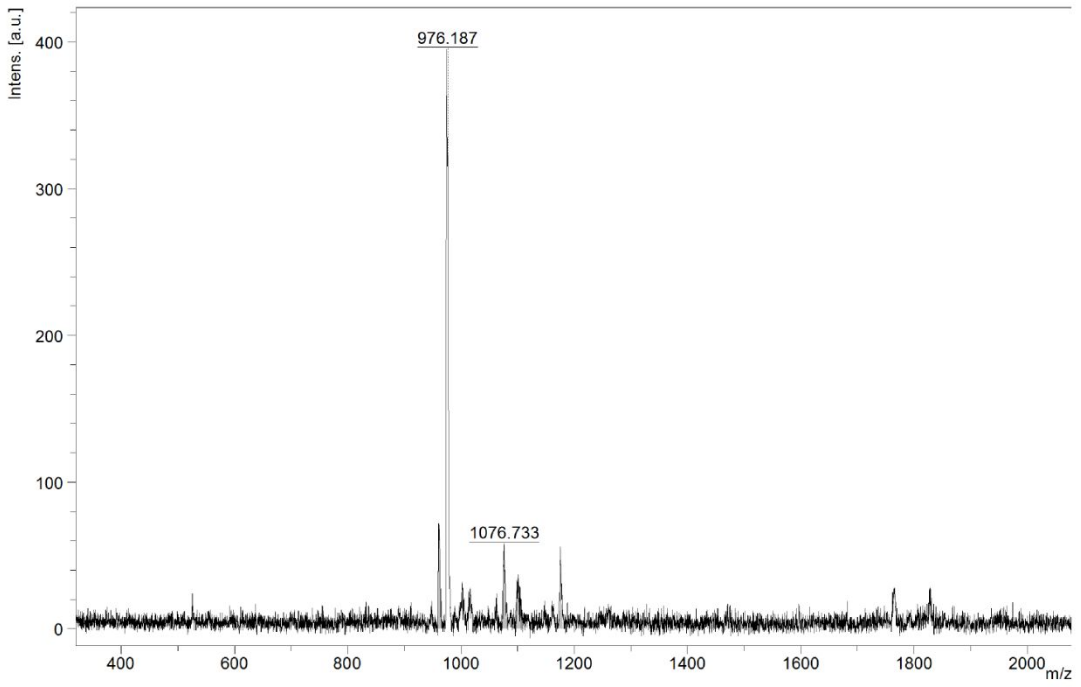

Figure S4. MALDI-TOF mass spectrum of **$CuPc(OHex)_4$** (matrix: DHB).

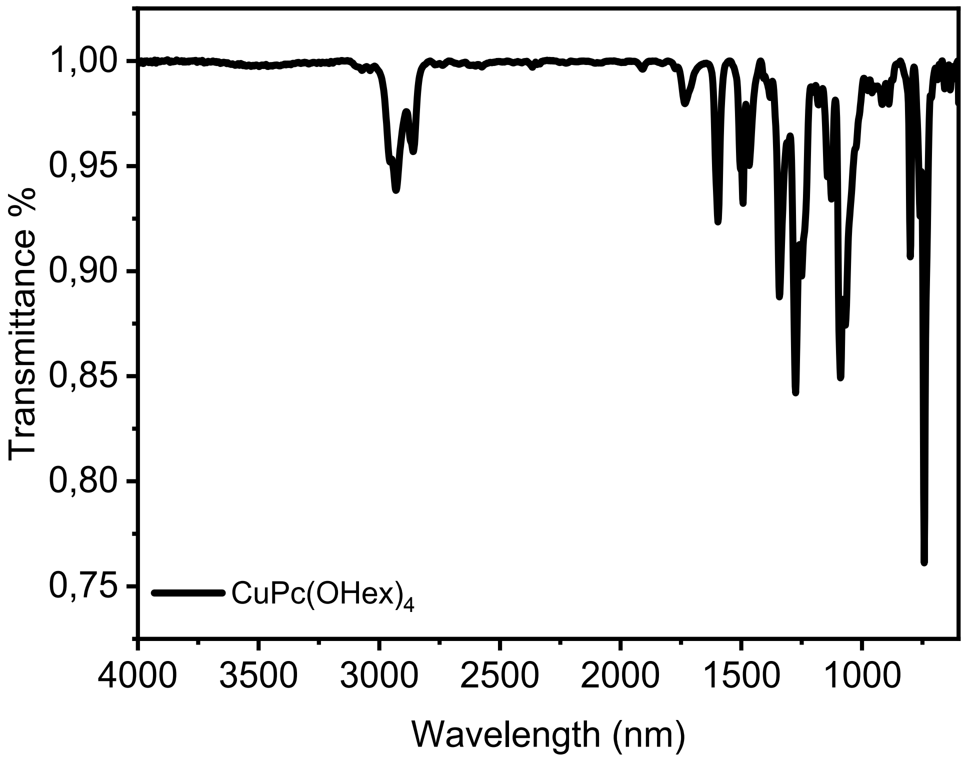

Figure S5. FT-IR spectrum of **CuPc(OHex)$_4$**.

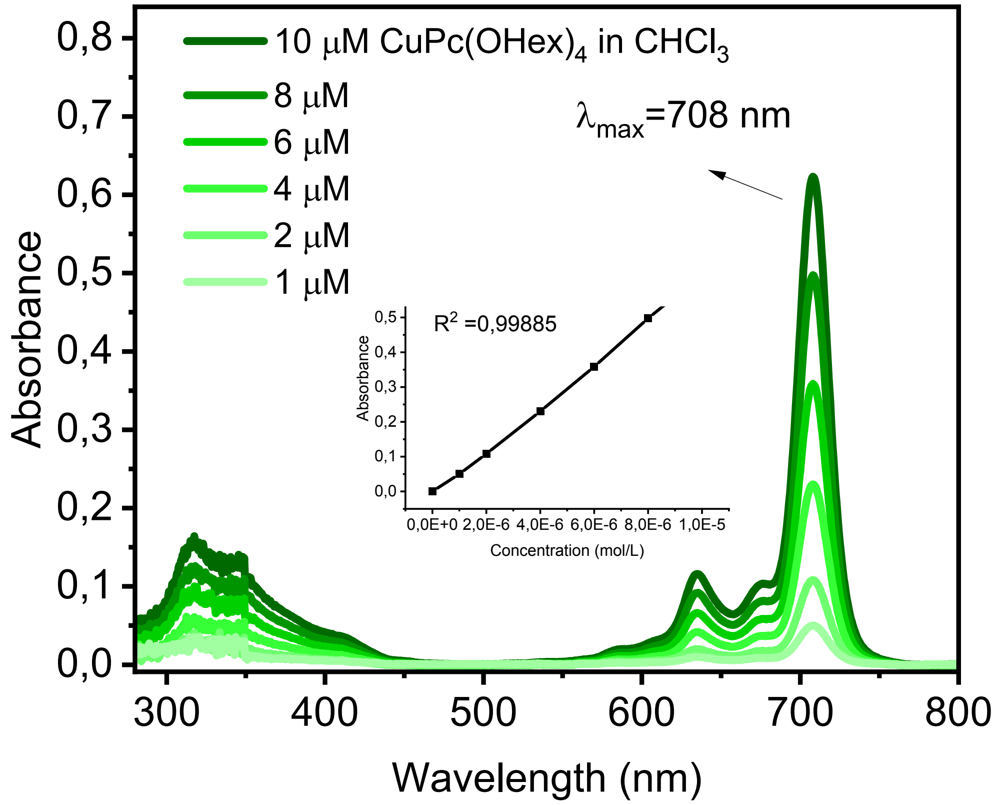

Figure S6. UV–Vis electronic absorption spectra of **CuPc(OHex)$_4$** (chloroform, 1-10 μM). Inset: Plot of absorbance vs. concentration at 708 nm.

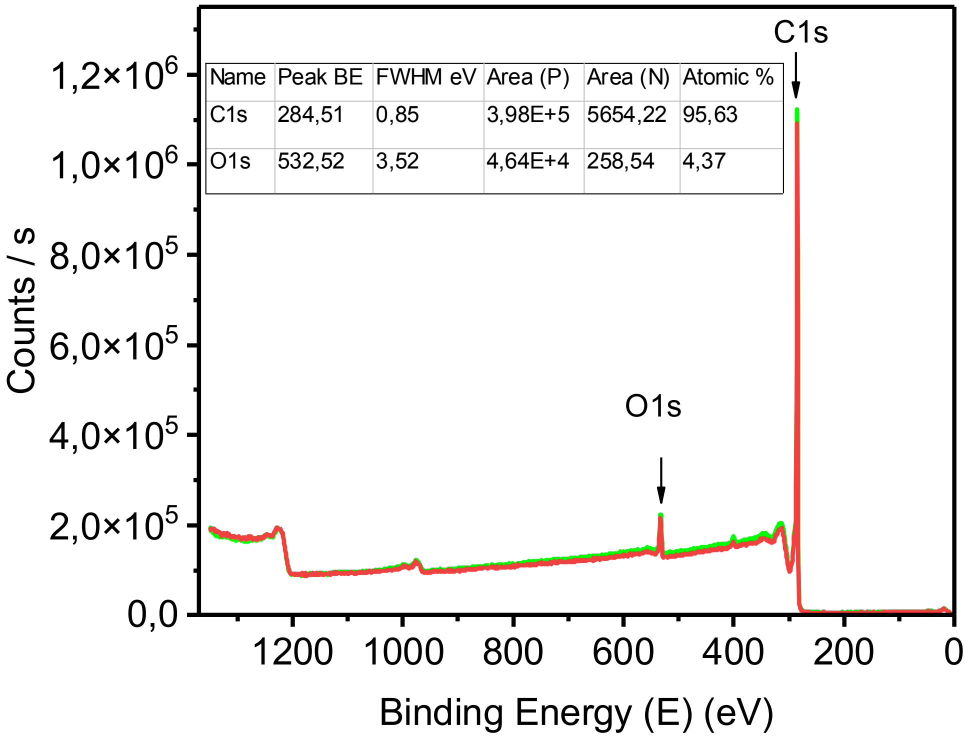

Figure S7. XPS survey of **rGO**.

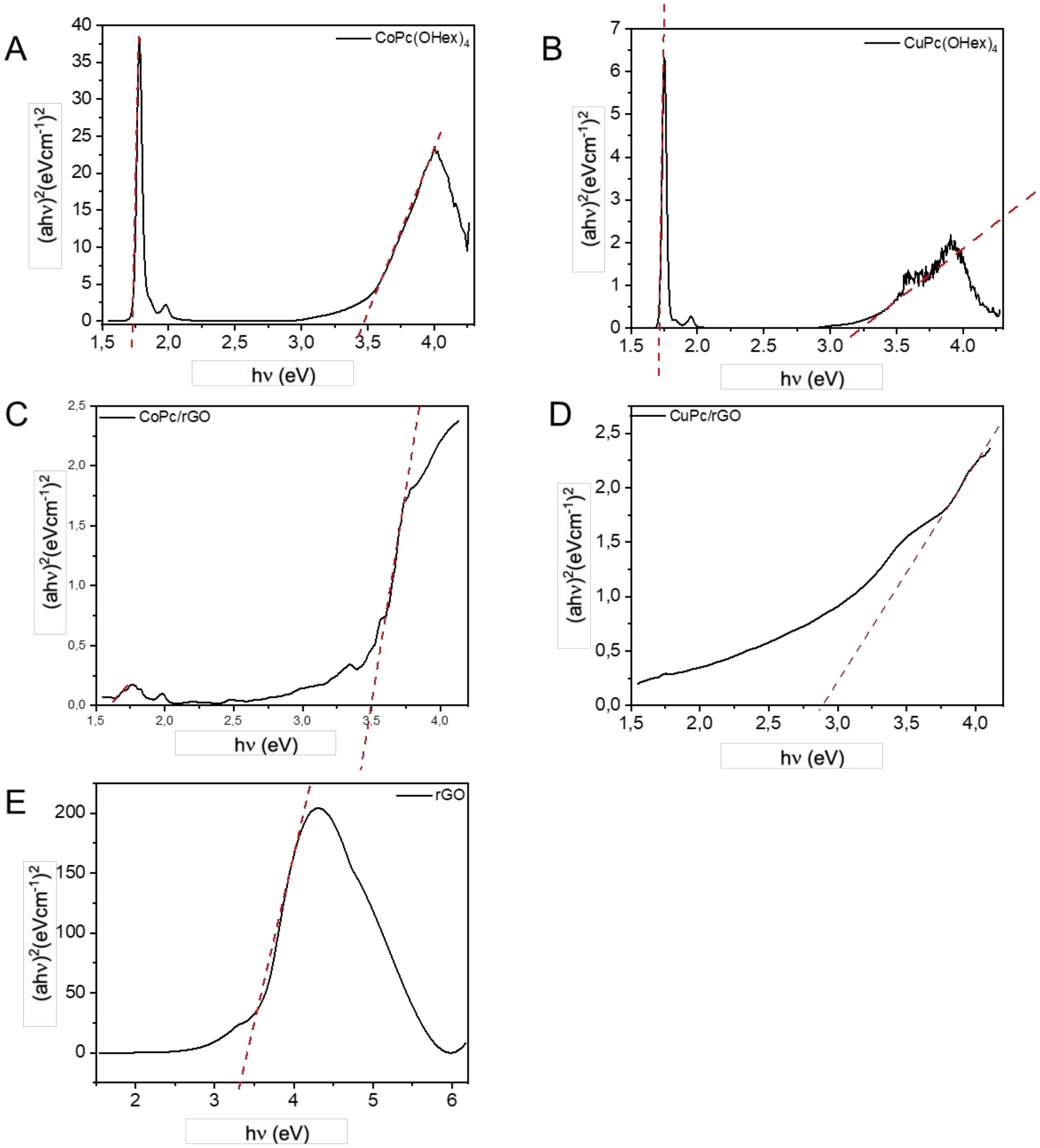

Figure S8. Determination of the band gap of A) **CoPc(OHex)$_4$**, B) **CuPc(OHex)$_4$**, C) **CoPc/rGO**, D) **CuPc/rGO,** E) **rGO**..

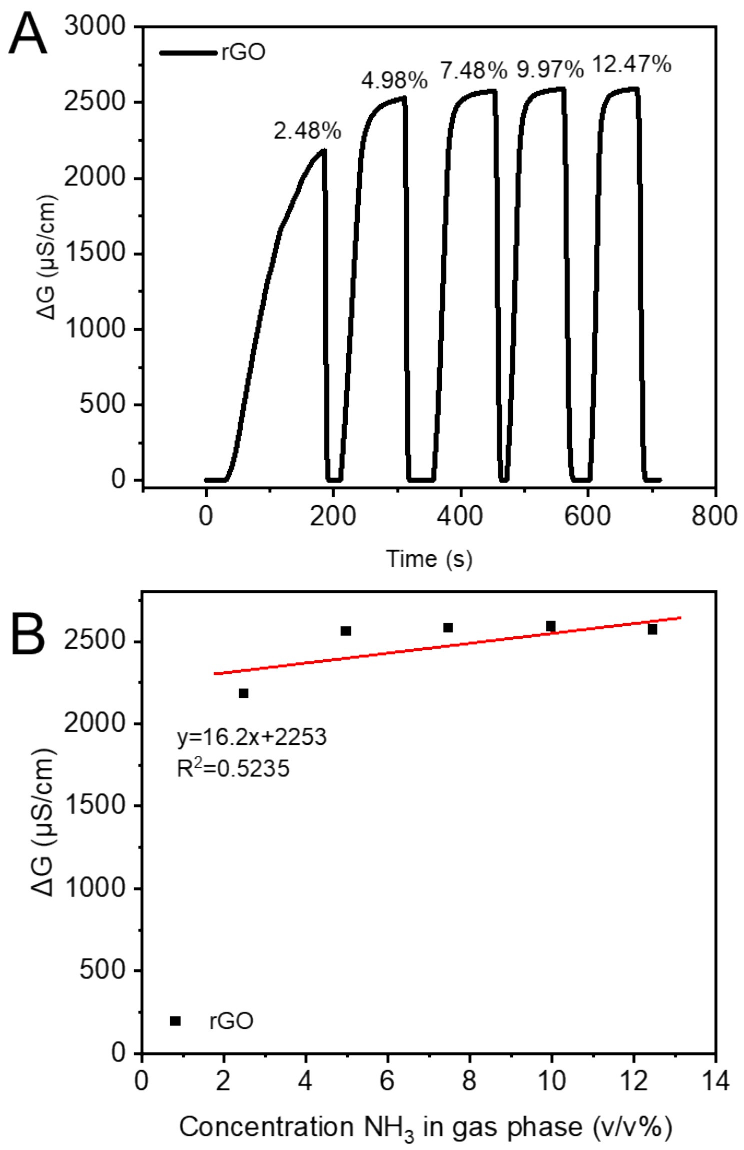

Figure S9. A) Detection of gas-phase concentration for various concentrations of ammonia with rGO sensor. B) calibration curve presenting the maximum value of resistance within the concentration of ammonia in gas phases with rGO.

Table S1. Equilibrium aqueous phase molar concentrations and gas phase concentration of ammonia

Volumic concentration (%)	Molar concentration in the aqueous phase(M)	Concentration in the gas phase (v/v%)
0	0	0
0.9	0.38	0.61
1.8	0.77	1.24
3.6	1.54	2.48
5	2.14	3.46
7.2	3.09	4.98
10	4.29	6.92
10.8	4.63	7.48
14.4	6.18	9.97
15	6.44	10.39
18	7.73	12.47
20	8.59	13.85
25	10.73	17.31

Table S2. Equilibrium gas phase molar concentrations of various gas at 25 ^{o}C per Henry's law constants

Volumetric concentration (%)	Methanol concentration in gaseous phase (v/v%)	Ethanol concentration in gaseous phase (v/v %)	Acetone concentration in gaseous phase (v/v %)
0	0	0	0
5	0.56	0.45	2.81
10	1.12	0.90	5.62
20	2.25	1.80	11.25
40	4.49	3.60	22.50
60	6.74	5.41	33.75
80	8.99	7.21	45.00
100	11.24	9.01	56.24

Table S3. XPS Binding energy values of nanocomposites and of their isolated components for $2p_{1/2}$ and $2p_{3/2}$ states

	$2p_{1/2}$ (eV)	$2p_{3/2}$ (eV)
**CoPc(OHex)$_4$**	796.53	781.81
**CoPc/rGO**	796.04	780.52
**CuPc(OHex)$_4$**	955.40	935.41
**CuPc/rGO**	954.77 (952.91)	934.74 (933.04)

Table S4. The electroactive surface of the nanocomposites and their isolated components.

	CV Fe^{2+}/Fe^{3+}			
	Ep_a (V)	ΔE_p (V)	Ip_a (μA)	A (cm^2)
**rGO**	-0.028	0.313	-104.4	0.28
**CoPc(OHex)$_4$**	-0.182	0.338	-100.2	0.273
**CuPc(OHex)$_4$**	-0.098	0.463	-90.55	0.24
**CoPc(OHex)$_4$/rGO**	-0.131	0.259	-120.2	0.33
**CuPc(OHex)$_4$/rGO**	-0.03	0.262	-119.5	0.32

Table S5. Fitting parameters of EIS data.

	R_s (Ω)	R_p (Ω)	CPE (μT)		W (Kσ)
			Y0	N	
**rGO**	550	475	1.46E-6	0.919	0.00236
**CoPc(OHex)$_4$**	563	566	7.75E-6	0.769	0.00212
**CoPc(OHex)$_4$/rGO**	532	239	7.02E-6	0.803	0.00259
**CuPc(OHex)$_4$**	445.96	921.1	8.83E-07	0.948	0.001514
**CuPc(OHex)$_4$/rGO**	467.21	286.72	6.82E-06	0.793	0.002432

Table S6. A comparison of various phthalocyanine-based sensors for NH_3 sensing

Nanocomposites	NH_3 LOD	Detection method	Response time (s)	Recovery time (s)	Ref.
1,8,15,22-tetra-iso-pentyloxy-CuPc/rGO	50 ppm	Resistance	no data	no data	[1]
tetra-β-(4-carboxy-3-methoxyphenoxy) CoPc/rGO (CPO)	3.7 ppb	Resistance	450	120	[2]
tetra-β-(4-carboxy-3-methoxyphenoxy) CoPc/rGO (cmpo)	16 ppb	Resistance	540	120	[2]
tetra-β-phenoxyl phthalocyanine cobalt	21 ppb	Resistance	420	78	[2]
tetra-β-(3-methoxyphenoxy) CuPc/rGO	23 ppb	Resistance	480	126	[2]
tetra-α-aminophthalocyanine Co/rGo	78 ppb	Resistance	225	250	[3]
tetra-α-iso-pentyloxy phthalocyanine copper/rGO	Down to 400 ppb	Resistance	364	115	[4]
tetra-α-iso-pentyloxyphthalocyanine nickel/rGO	Down to 400 ppb	Resistance	200	264	[4]
tetra-α-iso-pentyloxyphthalocyanine lead/rGO	Down to 400 ppb	Resistance	248	331	[4]
aminobenzyloxy)phthalocyanine cobalt/rGO	0.078 ppm	Resistance	225	250	[5]
substituent-free phthalocyanine cobalt/rGO	1.3 ppm	Resistance	80	80	[5]
rGO/4-pyridinyl-oxadiazole tetrasubstituted zinc phthalocyanine	82 ppb	Resistance	no data	no data	[6]

rGO/4-pyridinyl-oxadiazole tetrasubstituted cobalt phthalocyanine	140 ppb	Resistance	no data	no data	[6]
**CoPc/rGo** from **CoPc(OHex)$_4$**	1.0 ppm	Conductimetry	26.73	4.9	This work
**CuPc/rGo** from **CuPc(OHex)$_4$**	0.4 ppm	Conductimetry	68.06	2.11	This work

Determination of the electroactive surface area

The electroactive surface area of the various modified electrodes was calculated from the peak current using the Randles-Sevcik equation (Eq. 1).

$$Ip = 2.69 \times 10^5 n^{3/2} A D^{1/2} C v^{1/2} \quad \text{Eq. 1.}$$

where n is the number of electrons that takes place in the redox reaction, D is the diffusion coefficient, $v^{1/2}$ is the square root of the scan rate, and Ip is the peak current. The surface area (A) can be determined from the slope between Ip and $v^{1/2/}$.

References

[1] Zhou, X.; Wang, X.; Wang, B.; Chen, Z.; He, C.; Wu, Y. Preparation, Characterization and NH_3-Sensing Properties of Reduced Graphene Oxide/Copper Phthalocyanine Hybrid Material. *Sensors and Actuators B: Chemical* **2014**, *193*, 340–348. https://doi.org/10.1016/j.snb.2013.11.090

[2] Guo, Z.; Wang, B.; Wang, X.; Li, Y.; Gai, S.; Wu, Y.; Cheng, X. A High-Sensitive Room Temperature Gas Sensor Based on Cobalt Phthalocyanines and Reduced Graphene Oxide Nanohybrids for the Ppb-Levels of Ammonia Detection. *RSC Advances,* **2019**, *9*, 37518–37525. https://doi.org/10.1039/C9RA08065A

[3] Wang, B.; Wang, X.; Li, X.; Guo, Z.; Zhou X.; Wu, Y. The effects of amino substituents on the enhanced ammonia sensing performance of PcCo/rGO hybrids. *RSC Advances*, **2018**, *8*, 41280-41287. https://doi.org/10.1039/C8RA07509C

[4] Li, X.; Wang, B.; Wang, X.; Zhou, X.; Chen, Z.; He, C.; Yu, Z.; Wu, Y. Enhanced NH3-Sensitivity of Reduced Graphene Oxide Modified by Tetra-α-Iso-Pentyloxymetallophthalocyanine Derivatives. *Nanoscale Res. Lett.* **2015**, 10 (1), 373. https://doi.org/10.1186/s11671-015-1072-3

[5] Wang, B.; Wang, X.; Li, X.; Guo, Z.; Zhou, X.; Wu, Y. The Effects of Amino Substituents on the Enhanced Ammonia Sensing Performance of PcCo/rGO Hybrids. *RSC Adv*ances **2018**, 8 (72), 41280–41287. https://doi.org/10.1039/C8RA07509C

[6] Yabaş, E.; Biçer, E.; Altındal, A. Novel Reduced Graphene Oxide/Zinc Phthalocyanine and Reduced Graphene Oxide/Cobalt Phthalocyanine Hybrids as High Sensitivity Room Temperature Volatile Organic Compound Gas Sensors. *Journal of Molecular Structure* **2023**, 1271, 134076. https://doi.org/10.1016/j.molstruc.2022.134076
